# Counter-Discourse Activism on Social Media: The Case of Challenging “Poverty Porn” Television

**DOI:** 10.1007/s10606-017-9275-z

**Published:** 2017-05-16

**Authors:** Tom Feltwell, John Vines, Karen Salt, Mark Blythe, Ben Kirman, Julie Barnett, Phillip Brooker, Shaun Lawson

**Affiliations:** 1 Northumbria University, Newcastle upon Tyne, UK; 2 University of Nottingham, Nottingham, UK; 3 University of York, York, UK; 4 University of Bath, Bath, UK

**Keywords:** Social media activism, Counter-discourse, Grassroots activism, Critical discourse analysis; socio-political issues

## Abstract

In this paper we investigate how online counter-discourse is designed, deployed and orchestrated by activists to challenge dominant narratives around socio-political issues. We focus on activism related to the UK broadcast media’s negative portrayal of welfare benefit claimants; portrayals characterised as “poverty porn” by critics. Using critical discourse analysis, we explore two activist campaigns countering the TV programme *Benefits Street*. Through content analysis of social media, associated traditional media texts, and interviews with activists, our analysis highlights the way activists leverage the specific technological affordances of different social media and other online platforms in order to manage and configure counter-discourse activities. We reveal how activists use different platforms to carefully control and contest discursive spaces, and the ways in which they utilise both online and offline activities in combination with new and broadcast media to build an audience for their work. We discuss the challenges associated with measuring the success of counter-discourse, and how activists rely on combinations of social media analytics and anecdotal feedback in order to ascertain that their campaigns are successful. We also discuss the often hidden power-relationships in such campaigns, especially where there is ambiguity regarding the grassroots legitimacy of activism, and where effort is placed into controlling and owning the propagation of counter-discourse. We conclude by highlighting a number of areas for further work around the blurred distinctions between corporate advocacy, digilantism and grassroots activism.

## Introduction

The unsympathetic television depiction of welfare claimants living in low-income communities has frequently been dubbed *poverty porn*: a label that acknowledges the prurient and voyeuristic nature of such programming as well as the objectification of its subjects. In the UK, the popularity of programmes such as *Benefits Street*, *Benefits Britain*, *Skint*, and *The Scheme* has led to significant interest from researchers seeking to understand the public fascination with the genre as well as its role in reflecting current attitudes towards welfare, welfare claimants, and societal and socio-political issues more generally (e.g. MacDonald et al. 2014). One line of such research has conducted investigations into the content of social media discussions of poverty porn, e.g. both Brooker et al. (2015) and Doughty et al. (2014) highlight the high levels of mistrust, antipathy and even hysteria directed at the communities portrayed within the programmes. Notably, Brooker et al. (2015) describe the Twitter conversation around #benefitsstreet as being primarily “knee-jerk” reactions to the content and characters of the programme, with the majority of tweets being offensive and abusive towards them. More generally, boyd (2012) has drawn attention to the (as yet poorly understood) role that online digital media can have in propagating online cultures of such mistrust, suggesting that ‘hysteria isn't necessarily from on high, but, rather, all around us.’ In other words, no longer is hysteria delivered exclusively in a top down manner, for instance from broadcast media. It is now also propagated and reinforced at a grassroots level and is insidiously present in the social media streams that people encounter and absorb across the Internet and, in particular, on social media platforms such as Twitter and Facebook.

The effects of the everyday propagation of problematic portrayals of whole communities on social media are poorly understood, though in the worst case it is not unreasonable to assume that it could lead to a lack of tolerance, respect and inclusion, as well as fear, mistrust and their marginalisation. Set against a backdrop of austerity and deep government spending cuts, such effects could have severe offline implications for local and even for national social cohesion (Forrest and Kearns 2001; Parkinson et al. 2006). For instance, the spread of derogatory remarks on social media directed towards people claiming welfare benefits in the aftermath of the broadcasting of the first series of Benefits Street led to the UK Government Minister responsible for welfare claiming public validation of his swingeing austerity-driven reforms (Chorley and Chapman 2014).

Though it seems clear that online discussions of poverty porn alongside television broadcasts often propagate inflammatory and problematically provocative content, there is also scope to use social media in a more constructive manner in this setting. For instance, groups of activists could utilise the affordances of social media to provide a more balanced or counter viewpoint on specific issues. Indeed, one emergent focus of the literature on poverty porn has been the role that it might play as a catalyst for political activism; Hester (2014), for instance, speaks of ‘weaponizing’ the prurience of poverty porn to challenge prevailing attitudes. In other settings, social media has become an important political tool and is widely used by political parties, organisations and, indeed, activists to publicise, organise and mobilise their supporters (Gerbaudo 2012). Events such as the Arab Spring uprisings (Khondker 2011; Howard et al. 2011), the #Occupy movement (Juris 2012; DeLuca et al. 2012) and the Spanish (Anduiza et al. 2014), Greek (Theocharis et al. 2015) and Portuguese (Accornero and Pinto 2015) anti-austerity protests have motivated politics and communications researchers to reflect upon and study the communicative power of social media in moments of activism and social action. The HCI and CSCW community has also begun to build an understanding of many of the design issues stemming from digital activism and collective movements (e.g. Crivellaro et al. 2014; Asad and Le Dantec 2015; Foth et al. 2013; Wulf et al. 2013; Massung et al. 2013).

In this paper we extend this work by analysing the processes through which activists deliberately design, deploy and orchestrate online counter-discourse campaigns around socio-political issues. In particular, we explore how social media platforms are used in conjunction with more established forms of activism to generate a counter-discourse against the stigmatisation of communities portrayed through so-called poverty porn television. We focus on two examples of such activism that attempt to foster online counter-discourse against the dominant narrative of two series (or seasons) of the programme Benefits Street. Our work is motivated by a current lack of deep understanding of how digital activists design, deploy and subsequently orchestrate campaigns that challenge the dominant narrative of broadcast media. In studying these counter-discourse campaigns, we reflect on the effectiveness of the approaches used by activists and identify the ways in which existing social media platforms support activism work. Since we were specifically interested in understanding the counter discourse aspects of these activist campaigns we conducted critical discourse analysis (CDA) to understand the motivations of the activists and the online reaction to their activism. Using data from multiple social media platforms and through interviews with the activists concerned, we present an analysis of two case studies of responses to the Benefits Street programme: (i) the *Parasite Street* website and the subsequent discussion it promoted on Twitter, and (ii) a multi-platform social media campaign called *Positively Stockton*. Our findings highlight how activists tailor their understanding of success of their own counter-discourse campaigns to that of their target audience, that they specifically leverage the technological affordances of different social media platforms to control and contest discourse, and that access to networks of power and privileged spaces help amplify campaign messages. We also reveal issues surrounding the boundaries between activism, corporate advocacy and digilantism, and the considered usage of the affordances of social media by activists.

In the following section we first provide further background to digital activism and counter-discourse activism, as well as contextualising Benefits Street within the UK’s contemporary politics and broadcast media. Following this, we present our methodology and the findings from our critical discourse analysis. Finally, we discuss the key issues raised by the analysis, highlighting their implications for HCI, CSCW and social computing research.

## Background

In this section we discuss research related to digital activism, as well as the history and specifics of counter-discourse activism. We then present a summary of the background to the programme Benefits Street and describe relevant elements of the digital activism that have been mobilised against it.

### Digital activism and social media

Events such as the Arab Spring in 2010–11 and protests related to the 2008 financial crisis have been the driver for many activist groups, social movements and political campaigns, often played out over social media. Many of these are grass-roots initiatives, often instigated by dedicated activists as well as the citizenry. Traditional media, e.g. BBC (2012), has provided widespread coverage of these movements, as well as documenting their use of social media. Much work has been done to understand, typify and describe the way that activists are using technology and social media to propagate their messages, mobilise supporters and organise events. Studying social media usage during Spanish, Greek and US protest events related to anti-austerity and the Occupy movements, Theocharis et al. (2015) found that Twitter conversations at the height of these protests were predominantly used for disseminating information about the movement, rather than calls for action and organisation as previously thought. Poell and Borra (2012) studied activists at the G20 meeting in Toronto who tried to use Twitter, Flickr and YouTube as alternative channels for journalistic-type reporting as events unfolded in the city. They found that the majority of reports were from a small number of “insiders” and that contributions from “the crowd” were relatively small. This work reveals somewhat the difficulty activists have in mobilizing and motivating people from the wider population into political action.

DeLuca et al. (2012) explored how the Occupy Wall Street protest movement used Twitter, Facebook and YouTube as a means of bypassing traditional media and propagating their messages. Juris (2012) provides an ethnography of the way members of the Occupy movement used social media to engage and mobilise large numbers of people in discussions and protests. Furthermore, the nuanced understanding of digital technology by activists as a means for organising and discussing movements and messages is demonstrated by Anduiza et al. (2014), who surveyed demonstrators involved in the Spanish 15 M anti-austerity demonstrations. They found that small organisations, such as neighbourhood and town action groups and single-issue movements, coalesced around and utilised the digital media platforms of the *Real Democracy Now* group, a large “umbrella” group, as a means of furthering their influence. In having privileged access to the *Real Democracy Now* platform, they linked people and small organisations together into a larger collective, which in turn enabled the large scale, multi-issue 15 M demonstrations in 2012. This evidenced an understanding of the ways social media works and its limitations (i.e. many small Facebook groups will not gain much traction) by the activists, and knowledge that collaborating and shared access to platforms allowed them to achieve the same goals.

Tonkin et al. (2012) analysed tweets relating to the London 2011 riots around two decentralised social movements, one aiming to riot and cause criminal damage and the other to counter the damage caused by the riots. Both of these movements were organic in their creation, stemming from news coverage of the riots. While the majority of rioters used closed communication platform such as Blackberry Messenger, the counter-movement openly used Twitter with hashtags such as #BroomArmy and #OperationCupOfTea. In their analysis of 62,000 tweets sent during the riots, Tonkin et al. (2012) found that Twitter was primarily dominated by users countering the riots, and that those users would actively use the retweet functionality in order to “name and shame” other Twitter users who were suggesting rioting and criminal action. Similarly, Lotan et al. (2011) modelled the flow of information between key players during the 2011 Egyptian and Tunisian Revolutions. By classifying types of Twitter users, they were able to identify those who would amplify messages, or spread them across geographical areas. This, importantly, also revealed deliberate exploitation of networks of privilege, which bloggers and activists would target in attempts to gain traction and attention.

### Slacktivism

Somewhat in contrast to this work, the relative ease with which political and civic functionality has been implemented into digital platforms has led to the emergence of the concept of “slacktivism”. Initially intended as a positive expression of bottom-up, youth-led activism, the term has now adopted a more negative connotation, referring to activities that require little effort yet give the participant a sense of (self)satisfaction and political engagement. Morozov (2012, p.190), for instance, notes that the technological ease with which campaigns can be created using platforms such as Facebook has led to activist campaigns that reduce complex societal issues to issues solvable purely by social media, as they are based on ‘the assumption that, given enough tweets, all the world’s problems are solvable’. However, Christensen (2011) refutes the use of the “slacktivism” term, contending that it is used pejoratively to ‘belittle activities that do not express full-blown political commitment’. Christensen (2011) instead investigates the efficacy of online campaigns and services that have been branded “slacktivistic” in nature, and demonstrates that although it is difficult to identify positive off-line effects, existing literature suggests that digital political participation has a weak positive link with offline political involvement, and that there is no clear negative link with offline political involvement. While noting the need for further work, Christensen (2011) concludes that slacktivism is a harmless form of digital political participation that might at worst lead to increased awareness about political issues.

Conflicting views of the efficacy and value of “slacktivist” activities remain evident, however. Lee and Hsieh (2013) note that donations to specific concerns rise after participation with online petitions, whereas Schumann and Klein (2015) found interaction with slacktivist-type activities reduced the willingness to participate in physical, off-line actions. Importantly, Schumann and Klein (2015), p.318) also emphasise there is a complex underlying ‘relationship between low-threshold online and offline collective actions.’

### Counter-discourse activism

The concept of counter discourse has its roots in the work of Foucault (1970) (as outlined in detail by Moussa and Scapp 1996) who argued that when those who are normally spoken for and spoken about begin to speak for themselves, they create a *counter discourse*, which is an act of resistance to power oppressing them. Such acts of political resistance can manifest themselves in many different forms. Sanford (2001) explores the varied counter discourse created by Maya women in Guatemala in response to cultural oppression and state-sponsored genocide. For example, activists and community members used newspaper adverts to spread information about those murdered in massacres, and countered anti-Communist and anti-Mayan histories of violence.

Recently, it has been observed how social media and online platforms have supported new spaces for, and forms of, counter discourse. In the wake of the 2015 Charlie Hebdo shooting, Giglietto and Lee (2015) explored the creation of a counter hashtag, #JeNeSuisPasCharlie, in response to the #JeSuisCharlie Twitter hashtag. The predominant discourse of #JeSuisCharlie stated that freedom of speech was under threat by religious intolerance. Giglietto and Lee (2015) observed how the #JeNeSuisPasCharlie hashtag established a counter discourse, rejecting the original framing with one that both denounced the attacks but distanced support for the work of Charlie Hebdo. This counter discourse allowed Twitter users to express their own political identity without fear of disrupting social norms. In a further example, through their analysis of the #sealfie campaign, Rodgers and Scobie (2015) show how Inuit communities were able to contest a discourse established by a well-funded NGO. Countering the widely celebrated “#selfie” produced by Ellen DeGeneres at the 2014 Oscars, members of the Inuit community replied with their own hashtag, #sealfie. This was in response to DeGeneres’s support of anti-seal hunting campaigns, and allowed the Inuit to reject the discourse of “cruelty” and “exploitation” around seal hunting. Over the period of a few weeks, the hashtag received widespread coverage on traditional media, along with outpourings of support from other indigenous communities, thus demonstrating how grassroots counter-discourse movements can stimulate broader political conversations.

Clearly, there are many examples of activism using digital tools; moreover, the nature of their utilisation for campaign propagation and organisation is only just beginning to be understood. Furthermore the acknowledgement and exploration of slacktivist-type interaction through social media is a hotly-debated area. While these examples of counter-discourse activism highlight the role social media can play in the construction of such campaigns, this remains a relatively understudied subject. What is lacking in the literature is an understanding of how such counter discourses are designed, deployed and then orchestrated by activists to be spread and discussed among social networks. In order to address some of these issues, we present an exploration of the ways in which two digital activists engaged in generating a nuanced counter discourse in a deliberate manner across multiple social media platforms.

### Benefits street and the language of poverty porn

The Channel 4 series Benefits Street is one of the most well-known examples of poverty porn television in the UK. Initially broadcast in 2014 in the UK, the programme gained both popularity and notoriety due to its provocative portrayal of welfare claimants living in the city of Birmingham. The makers of the show took the opportunity to contrast the seemingly feckless behaviour of the show’s subjects with the austerity and welfare reform being endured by the larger population. An example of the provocative nature of the programme was the overlay of the Twitter hashtag #benefitsstreet on screen at controversial moments to, seemingly, motivate viewers to engage with the live back-channel of discussion about the show. This supported online discussion about the programme during live broadcasting, coalescing around a single hashtag. Brooker et al. (2015) provide a comprehensive exploration of the qualities of the “official” online discourse, performing an analysis of the #benefitsstreet Twitter online discussion. They noted two periods of tweeting activity, with the majority of tweeting occurring during broadcast of the programme (approximately 30,000 tweets per episode), and far smaller amount of tweeting when the programme was off-air. During broadcasts, tweets were predominantly ‘knee jerk’ reactions grounded in the content of the programme, with a general theme of offensive, abusive and judgemental language and statements, focusing on the appearance of those in the programme, their living conditions or their attitudes. For example:

“White d looks like she hasn't brushed her teeth since 19” [ibid, p6]

“She got no money for food and stuff but sits there with an iPhone 5s?” [ibid, p6]

However, Brooker et al. (2015) also noted that during the online discussion, predominantly when the programme was not being broadcast, there was an amount of conversation that attempted to provide alternative viewpoints to the dominant narrative of the programme. This frequently took the form of the sharing of external links to substantiate and refute claims made in the online discussion or in the programme, as well as individuals and groups sympathising with the programme’s characters, or disagreeing with the themes of the show. For example:

“C4 are using naive and vulnerable people to get higher ratings, exploiting their lack of education, media misrepresentation” [ibid, p7]

The website *Parasite Street* was explicitly identified by Brooker et al. in the online discussion during the broadcast of the programme as a commonly occurring link, with the tweet content looking to contest the overall themes of Benefits Street.

Based on this work, it can be seen that the qualities of the online discussion during the programme’s initial broadcast echoed the views presented by the mainstream right-leaning press: i.e. that welfare was a major, perhaps unnecessary and often unfair, burden to taxpayers and the UK economy. This framing, or narrative, of the programme, and the significant coverage in online and traditional media, was reinforced by commentary from politicians during the six weeks that the show was on air, with the programme and issues it raised receiving discussion in the UK Parliament (2014) as justification for welfare reform.

The rhetoric of poverty porn and welfare reform are deeply rooted in the media and political debate. Mooney (2011) describes the predominant rhetoric, termed as “Broken Britain”, as being rooted in a perceived breakdown in family unity. Attributed to teenage pregnancies and a reduced societal emphasis on marriage, this rhetoric contends that much of the population now depends on the welfare state, and is the cause of other social issues. Runswick-Cole and Goodley (2015) notes how public discourse has become:

“saturated with rhyming soundbite dualisms (shirker/worker; striver/skiver) and pejorative stereotypes of teenage mothers, feckless fathers, troubled families and fraudulent claimants”. [Cole 2015, p2]

Indeed, Slater (2014) examined how politicised “Think Tanks” published research articles which further perpetuated the concept of “Broken Britain”, which in turn circumvented engagement with the societal and state causes of poverty, directing gaze instead on the supposed dysfunction of family units. Slater posits that this is a politically motivated choice, and encourages support for austerity measures:

“the pages of policy reports and into public discussion […] welfare reform enthusiasts need a populist language in which to articulate this story of state and personal welfare failure. It is through the explosion of 'poverty porn' television that welfare discourses of political elites have become translated into authoritarian vocabularies.” [Jensen 2014, p2].

The company that produced Benefits Street, Love Productions, have produced a series of documentary programmes focused on austerity and poverty that have been both critically acclaimed by the media and the public and critiqued by segments of the press. In response to the accusation they are producing poverty porn, they defend their programme making decisions as exposing relevant issues in British society (as described by Plunkett (2014). However, Jensen (2014) counters this defence as “a pre-emptive sleight of hand”, asserting that “such programming is 'porn' in the sense that it aims to arouse and stimulate the viewer, to provoke an emotional sensation through a repetitive and affective encounter with the television screen” [Jensen 2014, p3]. At the time of writing, there have been two series of Benefits Street: series one aired in January 2014 focussing on the residents living in James Turner Street in a Birmingham suburb; series two aired in April 2015 and was focussed on Kingston Road in Stockton-on-Tees in the North East of England.

### Online counter discourse to benefits street

As outlined above, counter discourses are often established by those who feel their voice or position is not being represented by dominant discourse. Throughout the broadcast of Benefits Street there have been a number of counter discourses that have emerged from different individuals and groups challenging aspects of the shows dominant discourse. For the purposes of this paper we focus on two of these as case studies: Parasite Street and Positively Stockton-on-Tees.

#### Parasite street

**Fig. 1:**
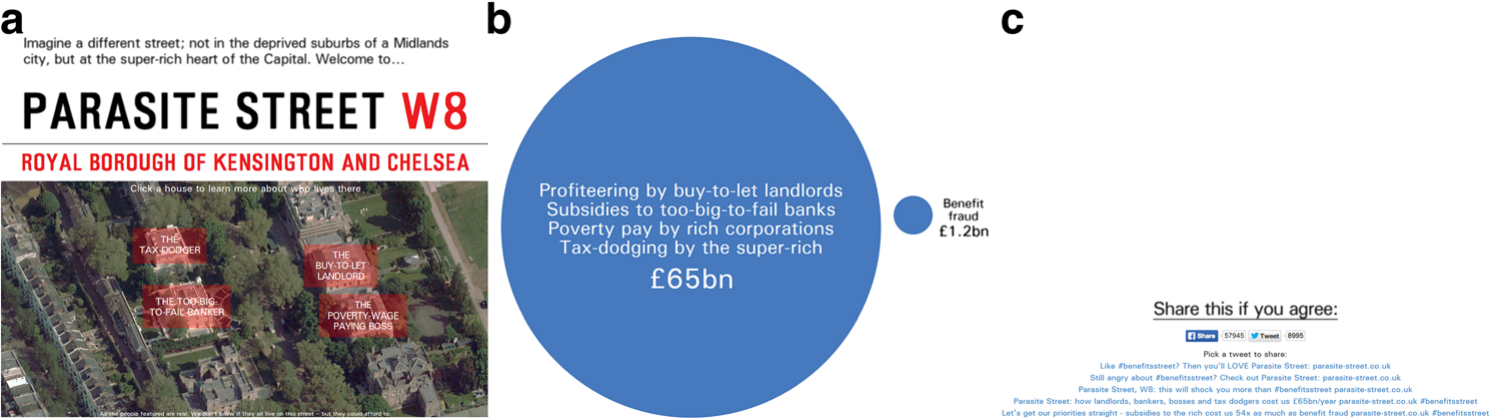
From left to right: **a** Landing page of Parasite Street website; **b** Visualisation of cost used on website; and **c** Prefabricated tweets and sharing functionality. © Stephen Reid 2014

Parasite Street (2014) is a website created in January 2014 as a response to the first series of Benefits Street. The website was developed and launched by Stephen Reid (SR), a self-titled “digital activist”, as part of the hacktivist collective “Undergr0und”. The website asserted that subsidies to the rich cost fifty-four times more than welfare fraud, and presented this through a short narrative backed up with visual graphs and other information. The website consists of only a single page which tells the narrative as the user scrolls down (Figure 1a). The page features a map of “Parasite Street”, modelled on a wealthy area of London, populated with interactive buttons. Each of these buttons produces a pop-up that outlines problems related to the UK economy following the 2008 world recession. For example, one of the “problems” referred to is tax avoidance: the process of legally reducing a corporation’s tax liability which is often discussed in the UK media and central government. Another button highlighted the issue of buy-to-let landlords: the process whereby landlords purchase houses specifically to rent out, which in some areas of the UK causes inflation of housing prices and pricing ‘normal’ buyers out of the market. Another refers to the problem of too-big-to-fail banking, noting how some financial institutions have a great deal of state involvement, which would cause great economic problems if they were to fail. Underneath the section outlining these problems, the website featured a simple graph showing the comparative cost of subsidies to the rich versus welfare fraud (Figure 1b). At the bottom of the webpage there was embedded the typical sharing functionality for Twitter and Facebook users. There was also a set of 5 pre-fabricated tweets that a Twitter user could click on to send (Figure 1c).

Alongside the website, the campaign utilised Twitter as its primary social media platform. The Twitter accounts held by SR and Undergr0und were used to promote the website on its initial launch. Two Twitter storms were organised using the “crowdspeaking” platform Thunderclap.it, which allows activists to coordinate large-scale tweeting efforts among their supporters to “increase [a project’s] social reach” (Wardle 2014). Each Thunderclap contained a pre-made tweet mentioning Parasite Street and the “official” Benefits Street hashtag #benefitsstreet, and were timed to coincide with live broadcasting of an episode of Benefits Street. Twitter users could sign up via the Parasite Street website to have their account participate in this Twitter storm.

#### Positively stockton-on-tees

The Positively Stockton-on-Tees (PSOT) campaign was launched in November 2014 in response to the commencement of filming for the second series of Benefits Street. The campaign was supported by Stockton-on-Tees Borough Council (the local government authority) with Mike McGrother (MM), a local activist and Creative Partner for the council, as a key organiser. Given the expectation, based on the first series, that Stockton would be portrayed negatively in the programme, the PSOT campaign was aimed at promoting positive stories about the town. The campaign also intended to “poke fun” at Love Productions, the producers of the show. The campaign ran from November 2014 and concluded in January 2016.

Unlike Parasite Street, PSOT was conducted both online and offline. The website (www.positivelystocktonontees.co.uk) was used as an archiving and blogging platform to record the events of the campaign. The website was structured into a short “About” page outlining the motivations behind the campaign, along with a page for each event associated with it. Each of these pages provided a 100-word summary of the event, along with images and videos. PSOT held accounts on Twitter, Facebook, Instagram and YouTube; however Twitter and Facebook were the primary platforms for communication. These accounts were used to share existing community events from Stockton, content from residents, and promote PSOT’s own events.

Between the start of the campaign and the first episode of Benefits Street 2, PSOT organised three major events. In February 2015, *Love From Stockton* involved MM visiting the Love Productions office in London to deliver Valentine’s gifts and perform a song. In February 2015 the *Great British Take-Off* (a satire of Love Production’s most popular programme in the UK, The Great British Bake Off) was released; this was a video directly questioning the methods of Love Productions and the portrayal of Stockton in the upcoming second series. Finally, *The Loudest Whisper* in March 2015 was a large community game of Chinese whispers involving around 5000 people. A troupe of clowns was hired by the PSOT campaign to carry the message between participants and to purposefully manipulate the message to remain the same throughout the event.

## Aims & methodology

In order to understand counter-discourse activism in relation to Benefits Street, we used these two prominent activist campaigns as case studies. Our aim was to understand the ways the activists identified problematic elements of a perceived dominant discourse, and attempted to counter-act these through a range of online and offline activities. As such, our research was oriented towards studying the ways the activists positioned their work in relation to Benefits Street and the nature of the discourse that underpinned their work.

To capture the varied approaches taken across Parasite Street and PSOT, we gathered a comprehensive dataset; for each of the case studies we collected data and discussion from social media and interviewed the individuals that orchestrated the campaigns. For the purposes of presenting our analyses, all social media comments that are not attributed to either of the campaigns as official accounts or a key person are anonymised and presented with pseudonyms (e.g. P1123) in accordance with British Psychological Society (2013) ethics guidelines.

### Data collected for parasite street campaign

The content of www.parasite-street.co.uk was collected (1 page), along with tweets from Twitter containing #parasitestreet from 15th January 2014 until 31st August 2015. This comprised of 360 tweets in total. This included tweets by @Undergr0und, and the Twitter account of SR, the creator of the site. There were in total 2068 tweets associated with the Thunderclap campaigns, but as these duplicated the original message, only the original message was included in the data set. A semi-structured interview was conducted with SR. The resultant audio was transcribed and included in the data set. A commentary piece written by SR for The Independent newspaper was also collected, along with webpages for the two Thunderclap.it campaigns.

### Data collected for positively stockton-on-tees campaign

For PSOT we collected: all 21 videos from the official YouTube account; all 950 tweets from the Twitter feed for @PositivelySOT, the official Twitter account; all 149 posts from the official Facebook page for “Positively Stockton-on-Tees”; and all 87 posts and related comments from the PSOT Instagram feed. The data ranges from December 2014 until August 2015. A semi-structured interview was conducted with MM, a main organiser of PSOT campaign. The resultant audio was transcribed and included in the data set.

### Critical discourse analysis

We conducted critical discourse analysis (CDA) to examine our datasets and draw out discourses and common themes. Our work was guided by Fairclough (2003), who defines CDA as the analysis of linguistics, power, and the continuity and structuring of discourse. The method is heavily rooted in the work of Foucault (1970) and maintains the view that power is discursive and does not exist in isolation from other communication and the world around it. As such, CDA is able to “perform the linking of social and political engagement with a sociologically informed construction of society” (Wodak and Meyer 2009, p.7). When analysing how discourses are challenged and countered, as well as understanding their impact on society, CDA allows us to understand the subtleties of language and specific acts of power performed through each campaign, as well as elucidating the technological features which enable or disable these actions. Therefore, CDA allows for a more nuanced understanding of the relationship between activists, technology and power structures.

During our analysis, we were specifically interested in the form of the counter discourses established by each case study, as well as the positionality and framing of these in relation to Benefits Street. We were also interested in the ways power was enacted through the various digital and physical platforms. Despite the textual focus of CDA, this methodology does not discredit non-textual content, and indeed in recent years there has been a growing appreciation of multimodal approaches to CDA that study how text, talk, images, film and other forms of media combine to enact and re-enact discourses (see Manchin and Mayr, 2012). More recently, discourse analysis has been used to study discourses on social media, including how power is legitimised and delegitimised within Iraqi political communities on Facebook (Al-Tahmazi 2015) and how politically charged YouTube videos and comments interact to promote alternate discourses around the same media (Way 2015). Building on this work, our use of CDA was in an analysis of various textual and non-textual content (interview transcripts, Twitter comments, Facebook posts, Instagram images and YouTube videos and comments) as a way of examining the work of the activist across these different platforms and how discourses were designed and deployed by them and engaged with by audiences.

In order to identify these features, we approached the data from a chronological perspective. The data analysis was performed by two researchers, and involved closely reading through the textual data and viewing associated video and visual data related to each case study. When reading through the data we were particularly interested in identifying key events that related to each of the activist endeavours (e.g. public events, significant postings, the broadcast of the television programme) as well as notable changes in the discourse over time. Through familiarising ourselves with the data in this way, we identified frequently occurring terms, words, expressions or visual tropes. From here the textual and non-textual datawas coded by the two researchers. Codes were created in an open-edned manner, with a focus on generating both descriptive and interpretative summaries of the data. Having coded the data, we compared the codes to one-another and clustered these around related and contrasting thematic frames. At this stage, we developed short memos to further summarise clusters of codes—these memos specifically focused on why and how specific language and linguistic features were used and the types of ideological and political goals they may have served. As such, our intention here was to “denaturalise” (Machin and Mayr, 2012) language and highlight ways in which events, people and entities are represented to meet particular ends. Following this, common themes were identified both within and across the data sets, from which we constructed a narrative based on excerpts of data related to each case study. We describe the findings from our analysis through these themes in the following sections.

## Analysis of parasite street campaign

In the following subsections we discuss our analysis of the Parasite Street campaign according to three prominent themes established during analysis: Positionality to Benefits Street, Configuration and Control through Online Platforms, and Propagation of Message and Engagement.

### Positionality to benefits street

The Parasite Street campaign was explicitly framed as a counter discourse to Benefits Street. The Parasite Street website predominantly contains indirect references to the show. For example the leading line on the website reads: “Imagine a different street; not in the deprived suburbs of a Midlands city, but at the super-rich heart of the Capital. Welcome to…” [Website, Parasite Street]. The invitation to the reader to imagine a different street assumes that they are already thinking of a street, and it is likely that they will have been guided to the website via online discussion around Benefits Street. Furthermore, it makes a direct reference to the show through articulating “not the deprived suburbs of a Midlands city”. This text is used consistently throughout the campaign. On social media, the framing of the activism as being in opposition to the dominant discourse of the show comes through direct and explicit use of the endorsed Twitter hashtag #benefitsstreet, for example: “#benefitsstreet is nothing, the real shit happens on #parasitestreet” [P12, 15.01.14, Twitter], and “You have heard of #benefitsstreet, check out #parasitestreet. A good response!” [P17, 15.01.14, Twitter].

The website and associated text are also positioned to align with and acknowledge the issues being addressed in Benefits Street. This is done using tweets that place Parasite Street in comparison toBenefits Street. Consider the tweet: “Parasite Street – much worse than #BenefitsStreet <website URL>” [@Undergr0und, 16.01.14, Twitter]. Here, the use of the words “much worse” invokes a comparison between the target and the source of the tweet, situating one in a more negative sentiment than the other. This implicitly acknowledges that the issue of welfare fraud is something to be negatively looked upon, but poses that the issues represented in Parasite Street as worse. In this way, the website acknowledges failings in the UK’s state welfare system – the issue Benefits Street sets out to illuminate – but contrasts this fraud with issues it deems as more problematic. This positioning is consistent with the motivations behind the campaign:

“It didn’t take much to switch the frame: there are scroungers in society but they’re not at the bottom, the real scroungers are at the top. Take the story but tell it in a different way” [Interview, SR]

The alignment between these two stories is facilitated by the existence of the Parasite Street argument in media already:

“Owen Jones [a British journalist who writes for the Guardian newspaper] had touched on similar ideas about tax-exploiters and buy-to-let landlords, and being involved with UK Uncut I was very familiar with the tax-avoidance side of things” [Interview, SR]

**Fig. 2:**
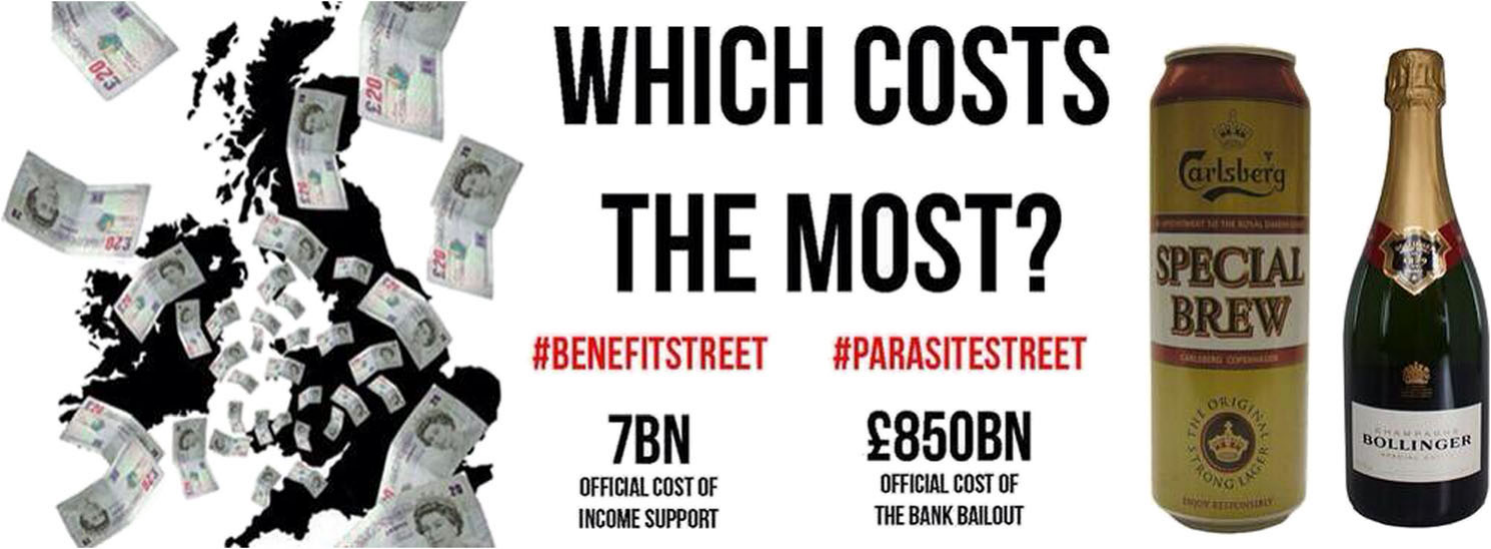
The London Economic’s infographic based on Parasite Street discourse. © The London Economic 2014

This framing was significant because it enabled Parasite Street to resonate with groups and individuals whose views aligned with those in this segment of the British press. This is evident in the data, with other groups creating infographics similar to Figure 1b in support of the Parasite Street argument. The first example of this, seen in Figure 2, was produced by a digital newspaper “The London Economic”, which tweeted their own infographic along with the following text “@Undergr0und @<anonymousCelebrityUser> Which costs the most? #benefitsstreet #parasitestreet” [@LondonEconomic, 16.01.14, Twitter]. The use of the @ symbol combined with a username, is an affordance of Twitter which will send a notification to those users, containing the tweet. This is known as a “mention” and is a powerful way to direct messages to any Twitter user. The infographic, very similar to the style presented on the Parasite Street website (Figure 1b), presents a similar framing of the issue to Parasite Street. Despite using both hashtags, the #parasitestreet wording is located with “£850BN official cost of the bank bailout”. This refers to the 2008 UK bank rescue package (a government action to stabilise UK banks), instead of the various “abuses of the super-rich” mentioned on the Parasite Street website. The London Economic describes themselves as a “digital newspaper with open and accessible views on business, economics, finance and politics” (The London Economic 2016) and as such it might be expected that their presentation of the Parasite Street issue is skewed towards finance and the economy.

A key stage in the campaign’s Twitter activity was when the usage of Parasite Street becomes dissociated from references to the Benefits Street show and hashtag. Two days after the release of Parasite Street, the first occurrence of the #parasitestreet hashtag without reference to Benefits Street occurs: “@David_Cameron You are a parasite we need to feed #ParasiteStreet” [P1282, 17.01.14, Twitter]. This tweet is an example of *frame bridging* (Snow and Benford 1988); the process of linking two separate, but congruent, discourses together. The tweet takes the literal connotation of the term parasite – a relationship where one gains at the expense of another – and applies it to the (then) UK Prime Minister. This was echoed in other tweets: “MPs waste lots of public money commissioning portraits! <url> #parasitestreet” [P1015, 17.01.14, Twitter] and “This MP, voted against this parliament bill, and now receives $100ks from oil companies” [P1095, 20.01.14, Twitter]. In these examples, two discourses are drawn together; the discourse established by Parasite Street that the rich are exploiting and taking money from the state, and the discourse established by the UK Parliamentary Expenses scandal of 2009, where some members of parliament (MPs) were exposed for claiming excessive – or even illegitimate – expenses.

SR commented on why he stopped publicising the Parasite Street campaign after the broadcasting of Benefits Street:

“During the last episode… it seemed a natural point to put it to one side. Detached from the Benefits Street TV show, it doesn’t mean anything straight away. It’s only relevant in a certain context.” [Interview, SR]

This statement creates an interesting contrast. At first, it is at odds with the ways in which tweets become used to bridge between separate discourses and take on new meaning. But it also sits in opposition to the continued usage of Parasite Street until 1st June 2015, 17 months after the creation of the Parasite Street website and hashtag. After mid-February 2014 the hashtag sees low frequency use (5–7 messages per week), with the majority being examples of frame bridging as described above.

### Configuration and control through online platforms

The Parasite Street website used carefully considered design decisions in order to frame and direct online discussion. The website was hosted on a private server, which allowed the creators a great deal of control over the content; but this also allowed them to avoid potential confrontation with a web hosting provider who may disagree with, or be asked by a television production company or broadcaster to remove the website due to the contentious nature of its content. There are no tools available for comment or discussion on the site itself, thus the website performs a one-way, authoritative communication with the readers, while allowing the message to remain clear and unchallenged [Website, Parasite Street]. There is no “official” hashtag for Parasite Street itself, the only mention of a hashtag throughout the website is to #benefitsstreet in the prefabricated tweets. As such, when reading the website it appears there is nowhere to direct comment or critique of the Parasite Street message to, apart from #benefitsstreet. However, immediately upon the publication of the website, Twitter users created #parasitestreet. For example, a user who is editor for a newspaper tweeted: “You’ll like #parasitestreet if you’re watching #benefitsstreet parasite-street.co.uk” [P4, 15.01.14]. Another user: “Some people on #benefitsstreet are stuck in low paid jobs, the businessmen live on #parasitestreet” [P1005, 15.01.14, Twitter] and another tweeting “Let’s get #parasitestreet trending people, if you like #benefitsstreet” [P1007, 15.01.14, Twitter].

The use of embedded social media sharing tools within the website itself allowed SR to monitor how people were engaging with the campaign, as well as channel their conversations to popular digital spaces that fed back information about the number of shares. He used these as a measure of success:

“I could see that at a certain point [the number of shares] started growing very quickly… if people are seeing it in their feeds, people are commenting and it’s entering the public mind in some way.” [Interview, SR]

Furthermore, the sharing functionality embedded into the website also displayed to visitors the number of times the Parasite Street page had been shared on those platforms (Figure 1c), which allows readers an insight into the frequency, temporality and digital space in which it was being shared. This gives a direct indication to the reader about how “hot” the topic of the website is. It can be assumed that a large number of shares indicates a lot of conversation going on around Parasite Street, and this might encourage readers to interact with the sharing functionality if they can easily see lots of people are talking about the website already.

The online discussion was carefully orchestrated using the sharing functionality provided. SR explained how drawing the reader’s attention to the opportunities to share the website with others was a critically important aspect of designing and producing the website:

“It’s very important to pay close attention to the share images… you’ve got half a second to grab people’s attention.” [Interview, SR].

SR also considered how Twitter and Facebook would display information about Parasite Street when it was shared by a user, and knew how to configure this specifically to maintain a consistent message. The pre-configured share information for Twitter and Facebook duplicated the first line of the website - “Imagine a different street; not in the deprived suburbs of…” [Parasite Street sharing text, Facebook.com] - as well as including a thumbnail image of the website. As noted in the previous section, by carefully structuring this shared information and using it across all platforms a consistent message was created. This was something which SR acknowledged as part of creating a “frame” for discussion:

“The intention was not to create a movement, but to create a frame… and as an opportunity for others to take that framing and talk in those terms.” [Interview, SR]

This framing is further evidenced in the language choices of the pre-fabricated tweets provided at the bottom of the website: “Let’s get our priorities straight – subsidies to the rich cost us 54x as much as benefit fraud <website-url> #benefitsstreet” [Pre-fab tweets, Parasite Street website], see Figure 1c for further examples. Here, the term “Let’s get our priorities straight” initially conveys a sense of action through “let’s get*”*, while “us” and “our” engenders an inclusive element to the message that aims to reach out to the reader. This phrase explicitly states the priorities of society are wrong, and that we need to fix them. It is hinted that this can be achieved, or at least elucidated, through reading the Parasite Street website. By ascribing emotion and politics, the tweets appeal to not only the reader of the Parasite Street website who is choosing which tweet to share, but to readers of the #benefitsstreet Twitter feed who will also see these tweets. The pre-fabricated tweet mechanism could be described as slacktivistic in nature. It relies on a simple interaction (click a button), and it is headed with the words “Share this if you agree”. Therefore, readers are encouraged to participate in sharing the Parasite Street discussion on social media as a way of expressing their agreement, and the content of the tweets does not incite readers to carry out any further political participation, such as attending a rally. This falls neatly into the definition of slacktivism as presented by Morozov (2012). However, SR’s intention here was to create a talking point around the issues raised in the website, without any further political aspiration. This stance is reinforced by SR during the interviews:

“The intention was not to create a movement, but to create a frame… and as an opportunity for others to take that framing and talk in those terms.” [Interview, SR]

The use of Thunderclap.it - a crowdspeaking platform (see Wardle 2014) that allows people to mass-share a tweet from their accounts at the same time - was also carefully considered and configured. The actual tweet used for the Thunderclap was:

“Parasite Street: see how subsidies to the rich cost us 54x as much as benefit fraud #benefitsstreet <Thunderclap URL>” [Thunderclap.it, 20.01.14]

As mentioned above, SR had purposefully chosen to re-use the exact wording from the Parasite Street website in order to maintain a consistent message. For SR, the use of Thunderclap was somewhat an unknown, but:

“it was a good lesson, you can maximise the impact of a Thunderclap by picking carefully the time of it… if you pick the right hashtags there’ll be lots of other people on Twitter reading about things.” [Interview, SR]

In his discussion of Thunderclap, SR demonstrated a thorough understanding of the way people interact with the Twitter stream, and explained how he used the Thunderclap platform as a means to inject the counter discourse of Parasite Street directly into the conversation of Benefits Street while the show was being broadcast. The fact that Love Productions used a Twitter hashtag for the predominant online discourse for Benefits Street allowed the Parasite Street campaign to directly inject their counter discourse into a highly-visited and highly-visible sphere of discussion. Critically, SR identified this was a sphere of discussion that could not be moderated by Love Productions:

“We timed to do them during the actual programme. A lot of people were tweeting… so that people searching for comments about the programme would then read these tweets and have a chance to think about things differently” [Interview, SR]

This allowed each Thunderclap to effectively hijack the official Benefits Street discussion and saturate the Twitter stream with their own counter-discourse.

### Propagation of message and engagement

Although predominantly guided to Twitter, the campaign also took advantage of other platforms with different power dynamics:

“Mentions of it [Parasite Street] popped up on various news articles, and I think Nick Clegg [then deputy UK Prime Minister] made mention of the framing, implying he had seen it. [There were a] number and type of mentions in other media channels as well.” [Interview, SR]

SR also authored a commentary piece in the Independent (a national centre-left newspaper in the UK) that presented and deepened the argument of the campaign. Similarly, he appeared in an interview on Russia Today UK to discuss Parasite Street, a channel which is known for covering topics outside the mainstream UK press, often critical of UK policy and government, and aligns closely with official Russian state discourse. The leveraging of these platforms demonstrated an understanding of the power available by propagating the campaigns message through traditional mass-media.

As well as using these more traditional routes to communicating the campaign in mass-media, to maximise the initial propagation of the Parasite Street SR also leveraged connections within his social network:

“There’s a network called NEON, I *…* emailed announcing Parasite Street and asking to tweet it […] I would [also] have given my friend at UK Uncut a call or an SMS saying ‘Hey, just my latest thing do you mind putting it up.’” [Interview, SR]

Indeed, within the first couple of days of tweets about Parasite Street those tweeting about it include the online editor of a major UK newspaper, online campaign groups and celebrities. For example, we have already noted how editors of UK national newspapers were engaged on Twitter, while also a well-known British celebrity with 12 million Twitter followers tweeted “Are we able to handle the reality? Welfare claimants are just a distraction from the real problem <Parasite Street URL>” [P2, 15.01.14]. This is a critical action for propagation as it allows access to credible and authoritative digital spaces, in the form of the Twitter feeds of campaign groups and newspaper editors. SR’s access to these spaces is afforded by his personal and professional social networks. Importantly, these kinds of discursive spaces are not necessarily accessible to grassroots activists.

The campaign was also designed to propagate messages quickly and concisely, so as to promote wide engagement and quick understanding of the matter at hand. SR explained how the website, its visuals and integration with social media platforms was tailored to present information in a way their target audience would understand:

“Not everyone has the time or inclination to read 800-900 word comment pieces… It's about taking out the key information representing it in a form that's consistent with people's minutely short attention spans in the Internet age.” [Interview, SR]

This is demonstrated in Figure 1b, which shows a visualisation from the website conveying subsidies to rich and fraud by the poor. Techniques have been used to position the issue of fraud by the poor, labelled “Benefits Fraud”, as smaller and less significant and in line with the overall Parasite Street framing. The large circle is labelled with four items, such as “Tax dodging by the super-rich”, whereas only one, “Benefits Fraud” is associated with the smaller circle. The font used to label the small circle is also smaller than that used to label the larger circle, and overall the visualisation is ambiguous because it does not mention whether the two circles are correctly represented to scale. While on initial viewing it may appear to be a simply constructed graphic, it is clear this carefully crafted visualisation is intended to quickly portray the message of the campaign, that the issues presented in Benefits Street are dwarfed in comparison with the issues around tax avoidance, buy-to-let landlords and other exploits of the rich [Website, Parasite Street]. The use of external sources to contextualise and reinforce the piece is described as underpinning the Parasite Street campaign:

“I value facts and I value information. There’s a sense that the stuff right wing political parties put out is fact-free. So by virtue of having some proper statistics it can pique people’s interest, and give an air of legitimacy.” [Interview, SR]

Through SR’s leveraging of an extensive network of activists, journalists and traditional media contacts, he was able to design and propagate a counter-discourse campaign that appeals to social media users in a way that directly contests and challenges people reading the #benefitsstreet Twitter feed.

## Analysis of positively stockton data

The second activist campaign we analysed was Positively Stockton-on-Tees (PSOT). As with Parasite Street, we divided our analysis across three common themes: Positionality to Benefits Street, Configuration and Control through Online Platforms and Propagation of Message and Engagement. Within each, we explored two key discourses; one contesting Benefits Street, and one that amplified Stockton-on-Tees.

### Positionality to benefits street

Unlike Parasite Street, the PSOT campaign positioned Love Productions, rather than the Benefits Street programme itself, as the target of the activism. Framing this discourse against the production company was an important strategic act whereby the target of the campaign became personalised to an identifiable group of individuals. This stance was made obvious by the first post made to the PSOT Facebook page, expressing the intention to “gently poke fun at the filmers [sic] of Benefits Street” [PSOT Facebook Page, 17.11.14]. This position is reinforced by the activists’ initial motivation:

“Let’s do something that’s really constructive criticism, you don’t get that in a petition… I don’t really feel that this is a campaign against Benefits Street, more to get people questioning things.” [Interview, MM]

This framing against Love Productions drove the creation of the Lovelier Productions “anti-brand”:

“I can’t match them for technical, or time… but I can do lovelier, I can do things nicer than them.” [Interview, MM]

As explored by Hollenbeck (2006), anti-brands are often social movements with a shared rejection of the corporate values embodied by a specific brand – for example, “Anti-McDonalds” and “McSpotlight” emerged in the early 2000s as anti-brands focused on highlighting the environmental problems caused by the multi-national fast-food retailer McDonalds. In a similar manner, Lovelier Productions is an overt attempt to create an anti-brand which stands opposed to Love Productions. The use of the word lovelier, while being a humorous play on the name of Love Productions, also produces an implicit qualification of Love Productions, stating the ambition that it is possible to have productions that are more loving to the places they describe and document in their shows. Appropriating the format of their name acts to draw comparison between the two, even though their production values and quality may not be on the same level technically.

Crucially, the anti-branding of Lovelier Productions was a critical part of the overarching PSOT campaign, and a key quality of the counter discourse of PSOT was the mimicry of the production company. The first of the PSOT events, *Love From Stockton*, sees MM visit the offices of Love Productions - specifically the office of Kieran Smith, their then Head of Factual Entertainment:

“I’m just here to deliver flowers [I said] … and he came out, I sang to him, they watched the film [Great British Take-Off], they said it was lovely, and thanked us very much [for the gifts].” [Interview, MM]

The act of delivering flowers and a Valentine’s card ties back to the anti-brand of Lovelier, as Valentine’s gifts are in contemporary popular British culture often associated with romance and love. It is also an important territorial act; by entering their private space he is perpetuating the sense that Stockton has been entered by Love Productions without the community’s approval. Furthermore, MM mimics their attitude:

“They asked me to come in for a meeting, but I haven’t got time for that. I’m playing the game. They’d never shown any interest in me before…” [Interview, MM]

The overarching message of *The Loudest Whisper*, the community Chinese whispers event, was, again, mimicking the attitude of Love Productions. Rumours had spread around Stockton that some of the footage filmed as part of the Benefits Street show was ‘re-enacted’ several times - thus calling into question the legitimacy of the programme as a documentary. The event referenced this rumoured manipulation of footage:

“The role of the clowns was to pass the message, but equally manipulate the message. The message isn’t in the words; the message is in the portrayal of the community.” [Interview, MM]

**Fig. 3:**
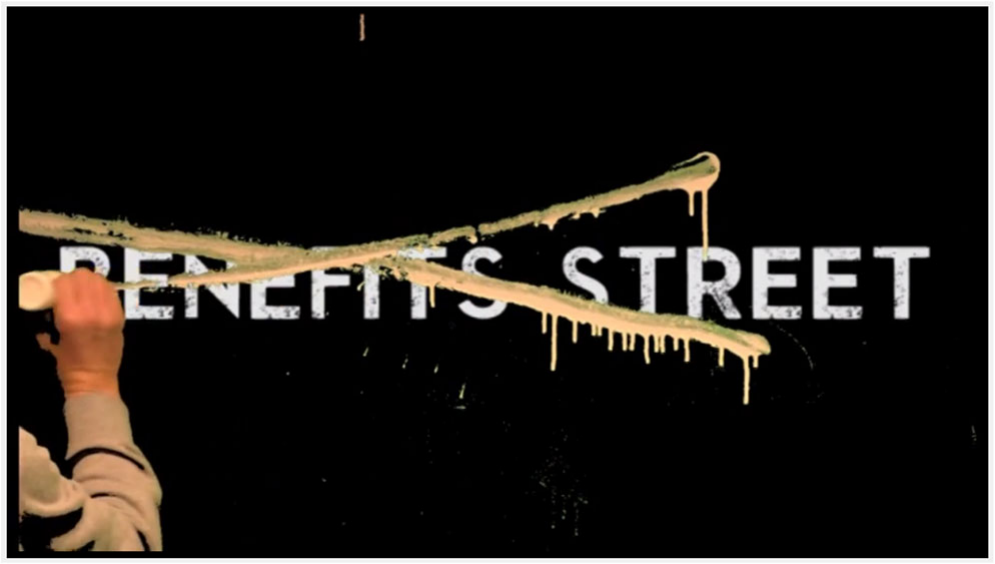
Screen capture from The Great British Take-Off. © Positively Stockton-on-Tees, 2014

This was further expressed in The Great British Take-Off video, where the campaign creates a powerful claim by appropriating and satirising the name of Love Productions’ most famous TV programme, The Great British Bake Off. In the video the Benefits Street logo can be seen being defaced (Figure 3). This is followed with a screen that read “It’s called Kingston Road”. In doing so, the video rejects Love Productions’ labelling of “Benefits Street”, and uses assertive and corrective language to relabel the street with its correct name. Overall, the video is overtly mocking, and confronts Love Productions about their representation of Stockton. Another scene in the video questions “Will you be showing everything that went on whilst you were here?” followed by “Or just what you chose to show”. This is then followed by a montage of images taken from the PSOT Facebook page and website, showing previous events, festivals, gatherings and firework displays. This is accompanied by the main musical theme of the Great British Bake Off. As the theme plays, it begins to distort and have electronic drums overdubbed, playing in a remixed style. The question “Will you manipulate?” is displayed, which is followed by clips of a controversy that surrounds an episode of the Great British Bake Off where Love Productions were accused of manipulative editing to make an incident in the show more dramatic, which led to emotional distress to one of the show’s contestants (see Deans 2014). By referencing an editing-based controversy, to which Love Productions never directly responded, they called into question their ability to provide a balanced view of Stockton on Tees.

### Configuration and control through online platforms

The PSOT campaign was focused towards the residents of Stockton, which initially drove the decision to use Facebook as the main means of communicating with the campaign’s audience. The Facebook page of the campaign very quickly got linked to other pages associated with local, news, information and events:

“there’s lots of little local sites [pages] like Norton News and Stockton Incidents. It doesn’t take long for people to say ‘It’s happening!’ and then myths begin before realities.” [Interview, MM]

**Fig. 4:**
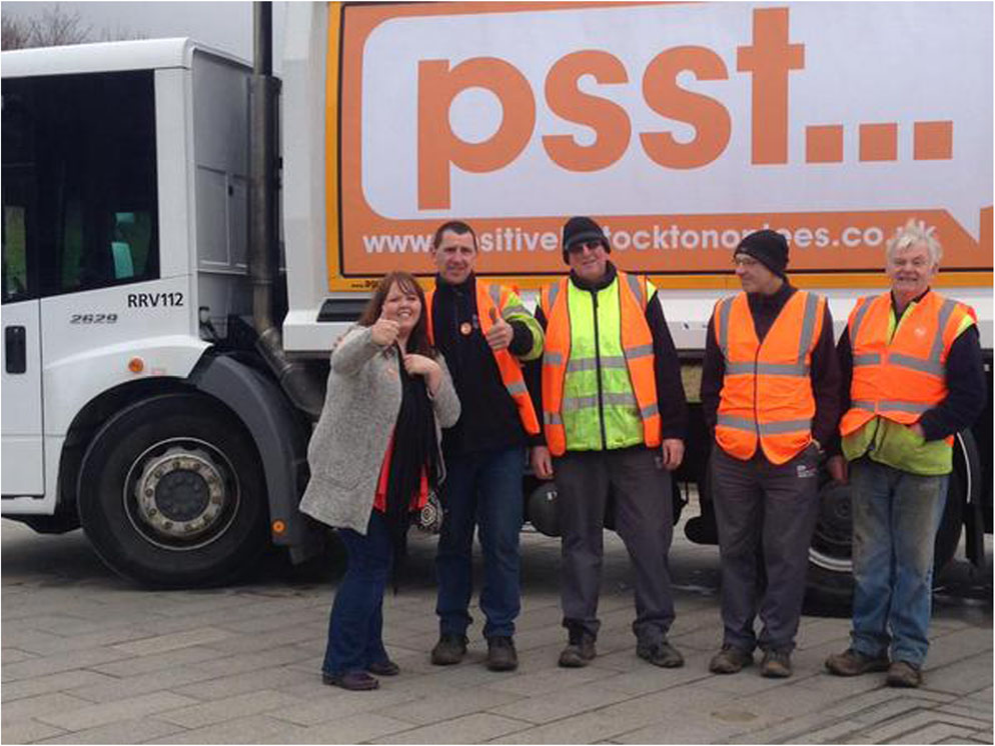
PSOT branded refuse vehicle. © Positively Stockton-on-Tees, 2014

Using Facebook as the primary social media was also seen to fit with the perceived dominant social media activities of the residents of the town. Situating the platform on Facebook would, as MM suggests above, allow the campaign to be involved in the discussion about Benefits Street 2, and allow them to counter myths and misinformation. In doing so, there was a sense that the activities of the campaign could be aligned with the daily social media activities of those who it wished to reach and participate in their events. The desire to engage only the local population in the campaign also manifested in the decision to advertise the campaign on a local refuse vehicle (Figure 4). The vehicle would be visible only to residents of Stockton, and was intended to direct them to the website and social media. By focusing the campaign on the town of Stockton, PSOT was able to produce a digitally and physically localised counter-discourse to Benefits Street, using Facebook groups oriented towards the local population as opposed to the wider public, and operate in physical locations that would only be seen by local people. This geo-localisation of the counter-discourse was in line with the organisers’ motivation to minimise the impact on the local community of the programme making, by allowing them to refute rumours and claims around the programme, as well as discuss the programme on their own, counter-discourse terms.

The PSOT campaign used careful orchestration across digital platforms to control the nature of discussion. YouTube was used purely as a means of delivering videos, with comments disabled, disallowing any interaction and discussion around the content uploaded on the site itself. This acted to direct viewers towards their main discussion platforms, Facebook and Twitter, thus reducing the digital spaces that might require moderation by the campaign organisers.

As noted, Instagram was primarily used for replicating content from the Facebook page, where the image shared would be identical to that used on Facebook and accompanied by a comment that would simply re-direct users back towards the website. For example, when promoting The Loudest Whisper event with an image of Psst… branding:

“#psst us for the first public session of #theloudestwhisper today? Be at #InfinityBridge #stocktonontees for 11.30am ready to start just after 12pm. Look out for the guys & girls in one of these funky hoodies - they'll keep you right! www.positivelystocktonontees.co.uk” [Instagram, @positivelystocktonontees, 15.03.15]

All posts provide a URL to the PSOT website, along with related hashtags (#psst, #theloudestwhisper). They also often used #stocktonontees to encourage residents to participate in the physical events, as well as to focus their posts to those from, and interested in, the town of Stockton-on-Tees.

The PSOT website also contained a page titled “The Story So Far” which took the form of a live social media feed aggregated from all of their official accounts. The language choice of “the story so far” transmitted a grassroots image of the campaign, which in part disguises its provenance as a local government supported and commissioned initiative. It also created a narrative to the campaign, linking individual events of the campaign together, which can be easily read by those not located in Stockton-on-Tees. This feed only contains social media posts from the official PSOT accounts, so the campaign is able to maintain tight moderation and curation of the content that appears within the “story”.

Critically, throughout their engagement with social media the PSOT campaigners were careful to avoid directly referring to the Benefits Street show. Throughout all of its activities, the official Twitter account never used any hashtags associated with Benefits Street, instead the activists chose to use their own hashtags for all content, predominantly #psst. By avoiding the wider Benefits Street discourse they were able to maintain a clear, largely PSOT dominated, online discourse that was distinct from the Benefits Street discussion. This further aligned with the organisers’ motivations to produce a localised counter-discourse for the residents of Stockton-on-Tees. The selection of the “#psst” and Psst… branding also gives the impression the campaign is operating underground and quietly, as the name is playing on the common form of quietly getting someone’s attention, e.g. “Psst… I have something to tell you”. By association, this gives the impression the campaign has something secret to tell readers, potentially something that others don’t want to be known. Importantly, this positioning of the PSOT campaign as a secret or underground initiative contrasts with the public funding of the work, as it might be expected that there would be more “official” council branding throughout the campaign.

When the second series of Benefits Street was first broadcast on the 11th May 2015, PSOT did not acknowledge the programme at all. Instead it used Twitter to retweet messages either mentioning the @positivelySOT account or that mention Stockton-on-Tees in a positive manner. An example tweet read: “If you want to see a rounded view, look at @positivelySOT #Stockton” [P52, 18.05.15, Twitter]. The authors of these tweets that were retweeted by the PSOT account either had a vested interest in Stockton-on-Tees itself (such as referring to Stockton as their hometown either in their account profiles or in the content of individual tweets) or were aware of critique towards so called poverty porn programming (for example, stating they were a politics researcher at a major university in their profiles). These retweets and interactions served to maintain a distance from the Benefits Street discourse while simultaneously engaging with supporters and co-opting their content within the counter-discourse.

It was notable that MM drew on a range of analytics and data to understand the reach and impact of the campaign:

“Actual views are about 50-60,000. But impressions I know it’s well over half a million. Everyone keeps messaging me saying it’s been shared all over the world” [Interview, MM]

MM regularly monitored who was commenting and engaging with the PSOT content. Doing this helped to signal important moments where he felt the campaign was having an effect:

“Their [Love Productions’] camera man commented on the video [on Twitter linking to The Great British Take-Off video on YouTube] and said ‘Ohhh, creepy’, so they know The Loudest Whisper is happening. That’s job done from my point of view.” [Interview, MM]

This informal discourse, via a comment, helped to signify to MM that people he and the campaign considered important, influential or part of the problem (i.e. Love Productions) were aware of his actions. This reaffirmed to him that their work was having some of the intended influence on what they do, even if in a small way.

### Propagation of message and engagement

The campaign established a strong discourse to amplify the qualities of Stockton-on-Tees, and specifically the community within it. To do this, the campaign actively solicited content from its followers:

“Hello to all our new ‘Like’rs! [sic] … Don’t forget to tell us why you love Stockton-on-Tees… why are you positivelySOT?” [PSOT Facebook page, 28.11.14]

Here, the use of the phrase “don’t forget” encouraged new supporters to tell the campaign about their feelings for Stockton. This was an invitation to share local pride, while also signalling that the campaign needed the contributions of local residents in order to function meaningfully. These contributions were further encouraged by a monthly competition, termed Positively Prizes, in which the campaign sought content from the local population. The campaign announced in December that it would be working with:

“Stockton Borough businesses over the coming year and handing out special prizes to some of the best pictures, stories, film and letters that we receive from you” [PSOT Facebook page, 15.12.14].

In February the first competition post appeared, with a meal at a local Stockton restaurant:

“Mohujos has very kindly donated a meal for four people in support of the Positively Stockton-on-Tees campaign… All you need to do is send us your photos and thoughts about why you are ‘Positively Stockton-on-Tees’” [PSOT Facebook page, 19.02.15]

The competition continued to run every month, with prizes such as meals, ice-skating lessons, and rowing-boat experiences. By encouraging supporters to share content with the chance to win locally-oriented prizes, this reduced the content creation burden for the organisers; it also resulted in contributions that were specifically focused on the town itself, thus creating a space for discussion around Stockton in the past, present and future amongst supporters.

The PSOT campaign also reposted content from elsewhere on Facebook, typically passing only brief comment itself. On the 31st of December, PSOT shared a post, originally from the page “Breaking News (Teesside)”. The post depicted a young girl who had cut off her hair for charity in aid of sick children. Alongside the shared post, PSOT commented: “Wow, this very generous little girl from Norton is an inspiration. Well done you lovely young lady” [PSOT Facebook, 31.12.14]. Acknowledging and praising this act helped to portray the campaign as being involved in the community, because it was sharing a post from a Facebook group dedicated to local news, but also as a campaign that harbours the value of charity and giving to others, by endorsing the girl’s actions.

There is, of course, a degree of tension in the manner in which the PSOT campaign positions itself however. While much of its counter discourse presents PSOT as a grassroots campaign, as already noted it is primarily a (local) government-run initiative. This tension is played out in the use of closed spaces for advertising (e.g. the side of a municipal refuse collection lorry, mentioned previously) as well as actions taken to associate themselves with officialdom. For example on 19th February 2015, PSOT posted a picture to Facebook of local councillors holding a Psst… sign. This was captioned “Great to see Cabinet members from Stockton-on-Tees Borough Council supporting the campaign!! #psst” [PSOT Facebook Page, 19.02.15]. This would suggest PSOT have been actively seeking endorsement by the local council, and are proud of receiving support from them. Interestingly, the single comment on this post reads “Mike said he wasn’t!” [PSOT Facebook Page, 19.02.15]. We would interpret this as meaning “he said he wasn’t involved with the council or supported by them”. The use of the phrase “supporting the campaign” in the PSOT is somewhat unclear, and only adds to the ambiguity of the council support for the campaign. The first video shared on Twitter and Facebook “Events So Far” is badged as created by the local governmental authority and highlights all the events that have occurred in the last year (2014) in Stockton. The somewhat ambiguous nature of who created the video muddies the understanding of who is running this campaign. A post showing the municipally controlled riverside lights in orange to support PSOT conveys the same ambiguity - the reader is not able to read whether this is official support by the local government (who control the lights), whether the campaign comes from the council itself, or whether the campaign have taken over the lights in a non-sanctioned manner. Moreover, the campaign uses spaces usually closed to grassroots activists, such as municipal refuse vehicles (Fig. 4) as a space for advertisement and an opportunity for a photo shoot. These acts are juxtaposed by some of the events conducted, such as Love From Stockton (the visit to Love Production’s offices), that convey the spontaneous, local-politics driven hallmarks of grassroots activism through their use of physical occupation. These actions build authority and legitimacy for the campaign as a grassroots movement. Indeed, MM frames himself as a local community activist, but works for the local council to run those events.

## Discussion

In the previous sections we have analysed how the two campaigns, Parasite Street and PSOT, positioned themselves in relation to Benefits Street, how they utilised platforms and technology to configure this positionality, and how they propagated messages related to their counter-discourse and sought engagement with public audiences. In this section we expand upon and discuss our analysis to extend and enrich the understanding of counter-discourse activism as it is relevant for the CSCW, social computing and human-computer interaction research communities. We structure this discussion around issues pertaining to (i) audiences and successes, (ii) control and ownership and (iii) power and privilege.

### Engaging audiences and understanding successes

Our analysis of Parasite Street and PSOT highlighted the ways in which both sets of activists, from the outset, had imagined audiences for their work; however these audiences were complex and, moreover, the ways in which social media and other digital services were used to reach them were multifaceted.

In the case of Parasite Street, the creator (SR) was driven by an aspiration to disrupt and inject alternative discussion into an existing Twitter stream around the first series of Benefits Street. His ambition was to reach out to and promote discussion amongst those who were already tweeting in relation to the programme’s broadcast; in doing so, he envisaged that those consuming and reflecting upon the programme’s dominant discourse would be confronted with an alternative narrative around state benefits. However, in order to reach this wider audience, the campaign had to make use of an initial, smaller, audience of Internet-savvy, politically-aware social media users who were likely already aware of the problematic politically-charged messages embedded in poverty porn television programming. These users were accessed primarily through SR’s existing personal online social network; this network was not only large (relatively speaking), it also included many well-known and influential left-wing journalists, political activists and bloggers. SR had access to this powerful and sympathetic audience and was able to speak directly to them through a counter discourse on the Parasite Street website that had an attractive ideological fit; this in turn provided the means to build a user base that was necessary for the crowdspeaking event. This, in turn, allowed the existing online discussion around Benefits Street to be disrupted, ultimately reaching the intended audience of social media users who were not necessarily already reflecting upon the values inherent in such television shows.

PSOT was perhaps more complex in its processes of engaging with its audience(s). In many respects, the ambitions of this campaign were similar to those of Parasite Street in that the campaigners wished to disrupt the existing discourse around state welfare, benefits and the othering of a whole town and its community through the promotion of an alternative narrative. As noted, however, much of the campaign’s social media activities, as well as the PSOT website, were focused on actively promoting positive stories and news of the local area. These stories were then propagated to those liking or following the PSOT campaign on Facebook and Twitter who were primarily people who identified with, or had some personal connection to, the town of Stockton-on-Tees. Therefore, while the primary work of Parasite Street was to rapidly and concisely convey politically-charged content as quickly as possible to a national audience, PSOT (on the face of things) presented itself as a slow-burning campaign that had local values and local legitimacy, carefully posing questions and communicating with what was imagined to be a primarily local audience, in order to build engagement and content over time. This was further supported through the use of offline promotional material related to the campaign, which often quite literally (as in Figure 4) spoke directly to those already living and working in the town. However, the focus on nurturing positive sentiments about the town served, perhaps intentionally, to deflect attention away from the campaigns’ ultimate - and quite subversive – objective; to discredit the creators of Benefits Street. Indeed, the three main events which were organised during the PSOT campaign explicitly targeted the creators, thus deviating from the focus on ‘positive stories’ to directly provoke the production company and challenge the tactics and methods used in the creation of poverty porn television. In many respects these interventions stood in sharp contrast to, and even contradicted, how the campaign otherwise presented itself on a daily basis; hence while the primary audience for the campaign was imagined to be local people who would share stories and take part in organised events, the ultimate ambition was to reach those seen to be creating the discourse to be countered in the first place.

Both sets of activists approached their respective campaigns with a deliberate goal; to create what we characterise in Foucauldian terms as a form of counter discourse. However, both campaigns also had differing audiences for their work; for Parasite Street the ultimate audience were those people discussing Benefits Street during its broadcast, while for PSOT it was those who identified positively with the town as well as, ultimately, the makers and distributers of poverty porn. The nature of the different audiences of the two campaigns led to two drastically different approaches to engagement. The use of pre-written, standardised text which explicitly eschewed overtly political language meant that the Parasite Street campaign purposefully appealed to those interested in simple, low-threshold activity as a means of participating in a cause – i.e., it was slacktivist in nature. While the impact of slacktivist campaigns remains contentious (as discussed in the background review), SR stressed that the primary aim of his campaign was to create a frame for discussion, and to propagate this frame as a way of talking about some of the issues being raised. In purposefully appealing to simple, low-threshold political activity (“click this button to share a tweet”) those who identified with the cause were able to spread the Parasite Street message unaltered, quickly, in a sphere where SR perceived there to be a dominant discourse around benefits and welfare. In the context of MM’s work on PSOT, however, such lightweight forms of interaction (e.g. sharing a post written by PSOT) were interspersed with more complex engagements where people with affiliations to Stockton were invited to contribute content, to offer positive news stories and to participate in organised events in the town.

A wider question remains as to whether counter-discourse activism work can be considered slacktivistic by nature. Both SR and MM put in a large amount of time and resource to conduct the campaigns, along with interacting with traditional media to ensure their counter-discourse was widely populated. The online-only nature of Parasite Street aligns the campaign more closely with slacktivism, with SR purposely reducing the campaign to low-threshold political interactions he is able to successfully, in his eyes, propagate the simple, but powerful counter-discourse of Parasite Street. On the other hand, PSOT features a strong online-presence, along with a complex configuration of offline relationships with the residents of Stockton-on-Tees in order to solicit participation in events, contribution of content for the campaigns, and interactions with town officials and residents to coordinate events. From these two case studies it is clear that low-threshold interactions can be a crucial component of counter-discourse activism campaigns, but that this is also dependent on the nature of the engagement and intervention. Therefore, it is unfair to describe them in the negative terms that have become associated with the “slacktivism” label, as the political engagement of organisers and participants, in some cases, can be far beyond low-threshold political engagement.

Regardless of the approaches taken to engage people in the counter-discourse, we might imagine that the perceived successes of the campaigns were measured by how far they reached their intended audiences. Our interviews with the respective activists reveal that the effectiveness of each campaign was measured using a range of methods by their creators, with (as might be expected) importance given to social media metrics such as ‘shares’, ‘likes’, ‘engagements’ and video views. Conversely, both stated they were not interested in the precise details and number of these social media metrics, and used them more as a rough gauge to whether their discourse was being shared. In this vein, they acknowledged that any evidence of wider groups or significant individuals engaging with campaign content was similarly, if not more, important as it was an indicator of how far and wide their counter-discourse was being propagated. In the case of PSOT, for instance, the moments where Love Productions themselves interacted with the campaign were valued as important signifiers of success, as were messages from people from outside of the Stockton area. For Parasite Street, the mention of the website by UK politicians, the invitation to author commentaries in mainstream media, and to be interviewed on television, were seen as critical successes. As such, although the quantification of success through measures of interaction and shares was viewed as important by the activists, a more nuanced understanding through interactions with supporters and observation of wider networks allowed them to evaluate their success in their own, often personal, terms.

This raises difficult questions for researchers whom are interested in understanding the effectiveness of activism and counter discourse on social media. In some instances, the outcome of the activist cause itself might be measurable, as noted on a small-scale by Crivellaro et al. (2014), and might even be related back to the activists’ actions; however, in more complex and wide-ranging cases (such as national issues around welfare as discussed here) this would seem to be unachievable. Related work by Potts et al. (2014) explores the way success might be measured, and the authors conclude that more work needs to be done around facilitating activists to leverage specific platform affordances in order to meet their campaign goals. Though elements of the activism discussed here have definite parallels with the social media marketing strategies frequently adopted by commercial organisations and corporations, conventional deployment of typical social media metrics when assessing brand and impression management (Hoffman and Fodor 2010; Peters et al. 2013) seem ill-equipped to provide deep insight into the impacts of activism.

### Control and ownership

In both campaigns, the presentation and manipulation of information aggregation on social media was important for positioning each campaign alongside the existing discourse. Our analysis shows how both campaigns used a central, conventional website as an authoritative space to present elements of their message in ways that could not be explicitly contested. However, when it came to engaging with and orchestrating their message via social media, each campaign had different approaches to maintaining control of their message.

Parasite Street was primarily concerned with explicitly positioning its alternative discourse alongside that which was seen to be the dominant reaction to the show. The campaign deliberately and explicitly chose to “hijack” the hashtag #benefitsstreet which was being promoted by the TV broadcaster - a strategy that has been used successfully by grassroots movements in the past to disrupt the marketing campaigns of commercial entities, corporations and government agencies, e.g. through the hijacking of the McDonalds’ Twitter promotion hashtags #McDstories (Mcfedries 2013) and #CheersToSochi (Pegoraro et al. 2014) and the #myNYPD law enforcement public relations campaign (Jackson and Welles 2015). In their analysis of the #CheersToSochi hijacking, Pegoraro et al. (2014) draw specific attention to the loss of message control by the original corporate entity - a clear objective of the Parasite Street campaign in this instance. Usage of the Thunderclap platform also allowed orchestration of large-scale tweeting at strategically important times, rapidly revealing the campaign to a wide audience in a firestorm (Pfeffer et al. 2014). Due to the deliberate use of #benefitsstreet and the timing of the Thunderclap alongside the start of the broadcast, Parasite Street was able to inject over 2000 tweets into the Twitter stream associated with Benefits Street at exactly the moment viewers would be looking at the Twitter feed. While with this comes the ‘danger’ of entering the unmoderated public discussion on Twitter, Parasite Street issued deliberately packaged pre-fabricated tweets and share text to set the terms by which the campaign would be discussed on social media.

The Parasite Street campaign’s practical attempts to create appealing tweets speaks to the general research challenge of constructing messages with a high likelihood of being retweeted or gaining momentum in a social network (e.g. as discussed by André et al. 2012; Comarela et al. 2012; Alonso et al. 2013). Understanding the impact of pre-crafted messages on social media has been explored empirically in mainstream politics; for instance, Bronstein (2013) found the levels of persuasion displayed in US presidential election candidates’ posts equated to more comments and likes. At the grassroots level, Juris (2012) found that appealing to mainstream, non-activist social media users was difficult due to the diffuse, non-centralised nature of the #Occupy movement and the subsequent lack of any agreed, actionable demands and political stances. In our analysis above, we suggest that SR’s use of social media sharing technology allowed the campaign’s message to remain unchanged. The simple pre-fabricated tweets were carefully crafted by SR to include evocative and persuasive language (“You’ll LOVE Parasite Street”, “Still angry about #benefitsstreet?”, see Fig. 1c), and the affordances of the sharing mechanism meant this message would not be modified (easily) by those sharing it. This addresses the issue identified by Juris (ibid) by enforcing a specific language and political framing into the tweets of Parasite Street’s supporters. The possibility of these messages being retweeted by other users in the Twitter network extends the reach of Parasite Street beyond the users interacting with the website, and maintains the original persuasive language crafted by SR.

In the example of PSOT, the maintenance of control over discourse on social media was enacted primarily through the use of language to communicate to their audience and through the choice of platforms with which to engage with these audiences. While PSOT did have an active Twitter account, this was primarily used to direct people towards the PSOT website or its Facebook page. On the few occasions where Twitter was used to invite discussion (e.g. when an episode of Benefits Street was starting to air), in contrast to Parasite Street, PSOT actively avoided using #benefitsstreet. In ways that echo the use of the hashtag #sealfie by Inuit communities, the PSOT campaign used its own hashtags as a means to make a conscious effort to distance themselves from the predominant online discourse around the television show. In a further means to instil control, PSOT used YouTube and Instagram primarily as ways to broadcast media related to the campaign; this was particularly explicit with their YouTube account which had comments disabled and was used as a way of enabling video content to be embedded on the main PSOT website or to be shared via the PSOT Facebook and Twitter accounts. Such usage of the video-sharing platform is now common by organizations - and referred to by Kim (2012) as the *institutionalization* of YouTube. Also, as noted, Facebook was the primary platform with which the campaign conversed with its audience; this appeared to be a further deliberate attempt to maintain rigid control of the discourse.

While PSOT presented a locally authentic identity and conveyed a spirit of being “bottom up” through the sharing of peoples’ own good news stories, discussion on the Facebook page was carefully controlled by only allowing followers to respond to posts made by a small group of people central to the campaign. As such, it purposely disallowed the public from proposing their own topics of discussion within the community page; this presented a context where it was seen to be fair to remove any comments and posts that might be considered to be deviating from any central and consistent message. Mascaro et al. (2012) studied this process of agenda setting by administrators of the Facebook page for the Coffee Party, an activist-initiated US political movement. Control over the social media discourse was implemented by allowing only administrators to initiate posts; although general group members were able to comment on these posts and engage in polyvocal discourse, the ability for moderators to set the tone of the discussion for each post, curate the entire page, and carefully erase comments that challenge the discourse of the political movement, facilitated strict top-down control and ownership of the Facebook discourse. This was echoed, although at a smaller and in a less politically explicit manner, by PSOT. It should be noted, of course, that the practice of carefully curating Facebook timelines so that they reflect a predetermined performance or message has been previously studied extensively for individuals (e.g. see Zhao et al. 2013; Zhao and Lindley 2014). Ammari and Schoenebeck (2016) recently discussed how Facebook groups are used to include and exclude certain voices from discussions around societal issues (in their case fatherhood and stay-at-home parenting) while Crivellaro et al. (2014) revealed that although Facebook seemingly provides a space for polyvocality in activism it can also be carefully moderated and managed by a privileged few, i.e. comments can be moderated out (or just simply proposed for moderation) - a practice that Crivellaro et al. relates to Hauser’s notion of ‘gentle violence’ (Hauser and McClellan 2010). It should be noted that platforms themselves play a key role in facilitating or restricting this process, with the use of “Terms of Service” enforcement by Twitter and Facebook to suppress anti-government activism during the Arab Spring being studied in detail (Youmans and York 2012).

Both campaigns therefore raise further questions around their attempts to take and maintain control and ownership of the online discussion; these include issues around the practices of curating individual messages and aggregated timelines but also around the power, governance and inclusivity of their campaigns.

### Power and privilege

While both sets of activists configured their work as primarily bottom-up and grassroots in-as-much as they were contesting dominant discourses being communicated in a top-down manner by media organisations and other powerful entities, both of the main protagonists had access to networks of power not normally available to grassroots movements.

For example, Parasite Street utilised a mailing list to access a loosely connected set of activists and organisations considered authoritative and credible when publicising the campaign. Previously, Juris (2005) in their analysis of anti-globalization activist networks also describe how the “creation of broad umbrella spaces, where diverse organisations and collectives converge around common hallmarks while preserving their autonomy” allows activists to loosely organise and cooperate. SR’s involvement in the hacktivist collective Undergr0und, along with his reliance on the activist network NEON and the UK Uncut social media account, are clear examples of such loose activist networks. UK Uncut defines itself as a “grassroots movement taking action to highlight alternatives to austerity” (UK Uncut 2016), and is aimed at countering the UK government’s austerity programme. Parasite Street, on the other hand, is aimed at the abuses of the super-rich and the problematic depiction of benefits claimants on TV. Since these political aims are ideologically related, SR was able to publicise Parasite Street on UK Uncut’s Twitter feed due to its operation as an “umbrella space” for members of the activist network. However, this is a privileged, closed space that is unavailable to many. Later in the campaign, Parasite Street even utilised traditional broadcast news media i.e. extremely powerful modes of communication unavailable to the majority. Though PSOT, in contrast, localised their audience, they also had privileged access to platforms, networks and services, such as placing advertising on local government refuse vehicles and in print. The former is a completely closed space, exclusively reserved for use by local government, and the latter is generally financial costly to utilise and publicize.

This privilege serves to muddy the definition of both campaigns as grassroots and activist in nature. Similar muddied terrain, in which a seemingly bottom-up campaign has obfuscated beneficial links to official power structures, has been explored by marketing scholars such as Beder (1998) via the term “astroturfing”: a grassroots movement created or supported by a company or organisation who may utilise “specially tailored mailing lists, field officers, telephone banks and the latest in information technology” in order to create a grassroots movement without any legitimation from the public. The term is heavily loaded as scholars often use it to denote subversive, malicious actions by corporations; for example, Beder describes how electricity companies attempt to influence legislation through the use of astroturfed advocacy groups. Similarly, Mix and Waldo (2015) raise awareness of the ethical implications of NGOs and corporations using astroturfing to influence democracy. Marketing researchers have also studied how brands on social media are promoted by seemingly “activist influencers” (Booth and Matic 2011) who are often covertly puppets of commercial organisations. For instance, organisations may identify social media users who may have large reach (such as someone with thousands of Instagram followers) and who are tangentially related to a product, before contacting them directly to advocate, covertly, for a brand. As such, in many respects the selection of activists by local government councillors in Stockton-on-Tees might be considered somewhat analogous to these examples in that MM was targeted to influence the discourse around Benefits Street series 2, and was seen as a local “celebrity” with influence. However, whilst the exact motivations of the councillors are unknown, it can be imagined they are poles apart from the motivations that drive more typical commercial users of activist influencers or astroturfing techniques. Nor should we conclude that MM was unaware of any aspect of his own role in the campaign.

An alternate view of MM’s involvement in PSOT, and even the use of well-connected activists and celebrities by SR in Parasite Street, is that it was a deliberate and intentional leveraging of followers or fans for a political cause. Bennett (2014), for instance, describes how Lady Gaga motivates her fans through social media to take part in her chosen activist causes. However, such use of privileged digital media spaces (e.g. through mobilisation of hundreds of thousands of Twitter followers) further blurs the boundary between activism and “digilantism” (digital vigilantism). Numerous examples exist of the leveraging of Twitter followers in order to spread views or incite action (see Associated Press 2016), and research studying the Reddit investigation of the Boston Marathon Bombings notes the potential for digilantism to provide positive civil investigation; however, the literature also warns of the susceptibility of the process to give way to speculation, leading to potentially devastating societal effects (Nhan et al. 2015).

Finally, though Nielsen (2013) observes when discussing #Occupy that the majority of online activism still uses ‘mundane internet tools’, he also reminds us that the use of such tools also limits participation and risks exclusion in itself. The PSOT campaign can be seen to be attempting to address this issue of exclusion by taking a multiplatform approach to propagate the campaign message. On the one hand, the campaign actively uses “mundane internet tools”, such as Facebook, Twitter and Instagram, in order to interact with a population who have ready access to social networking and prerequisite technology. It further leverages this techno-privileged audience by asking for them to contribute content using their own media (“Submit your photos, videos, etc.”). In contrast, the campaign also conducts work in physical spaces through events such as the Loudest Whisper, and locally-oriented advertising. However, in both cases outputs and further information of the events and about the campaign in general are situated on websites. Parasite Street emphasises this problem even further, as the primary focus towards just online social media platforms excludes those who do not regularly use these, do not engage in live tweeting practices, or do not have ready access to the required technology. Whether this somewhat exclusionary focus has a detrimental effect on either campaign is not clear, but the power afforded to each activist through the selection and placement of content in digital spaces has considerable influence over participation, and subsequent interaction with the campaign. While research on digital civic engagement and action has begun to pave the way for less-privileged digital forms of engagement (see Vlachokyriakos et al. 2014), the warnings of Nielsen (ibid) remain prescient not only for activists when designing campaigns, but for academics as a venue for further study.

## Conclusion

This research set out to explore the way that activists deployed and orchestrated activist campaigns focused on presenting a counter-discourse to an established, dominant discourse. Specifically, we analysed two campaigns surrounding the UK TV programme Benefits Street - a so-called poverty porn series that attracted widespread engagement by TV viewers, yet was criticised for stigmatising and othering people claiming welfare benefits. We used critical discourse analysis as a method to unpack the complex relationship between activists, technology and the public. Our work builds on a relatively small body of research focused on understanding the creation and deployment of counter-discourse campaigns by activists and our findings reveal that activists utilise an understanding of social media platforms and tailor their campaigns towards specific target audiences. Furthermore, while they utilise quantitative social media metrics such as shares and likes to measure success, they hold anecdotal feedback with equal regard, such as receiving mentions by traditional media and politicians. Our work has also revealed the leveraging of technological affordances of different social media platforms by activists as a means to control, contest or even own a discursive space. This is done through exercising different strategies in order to direct discussion to a particular space or use tools in a way so as to directly contest an existing discourse, such as guiding users to a moderatable space (as in PSOT) or taking advantage of an unmoderated platform to inject a counter-discourse (as with Parasite Street). We have explored some of the complexities of power and privilege that influence the creation, propagation and engagement of counter-discourse campaigns. Utilising different power modes of communication, either through the Twitter account of a celebrity, as with Parasite Street, or the side of a local government refuse vehicle with PSOT, campaign messages were placed in spaces that could only be accessed through an existing privilege. This, therefore, also raises tensions around the ambiguity of those grassroots legitimacy of activist campaigns that have access to power.

Our analysis has identified several areas that provide opportunities for further exploration by researchers in social computing, human-computer interaction and computer-supported cooperative work. The means for understanding and measuring the success of digital campaigns, online movements and activist campaigns are only just beginning to be explored (Potts et al. 2014). It is clear from our own work that activists use a mixed-methods approach when trying to understand the success of their campaigns, and subjectively appraise anecdotal feedback to measure its impact on the campaign. As such there are opportunities for researchers to understand more fully the spectrum of methods used by activists to measure their success. Further research is also needed into the utilisation of the technological affordances of different social media platforms in order to control a discourse. By examining the power inherent in the technical affordances of platforms we were able to elucidate the way they can be controlled, despite the majority of social media presenting itself as open and participatory platforms for discussion. The leveraging of these affordances by myriad groups, such as activists as studied here, through to far-right political groups, is currently poorly understood. Due to the complex and far-reaching effects that this kind of discursive control has on society, further research in this area is certainly needed. There are also opportunities for further research around the use of privileged access to digital media spaces by powerful social actors, such as celebrities and activists, in order to propagate and encourage engagement with a campaign or cause. The fuzziness caused by a variety of complex power relationships, between activism, digilantism and grassroots activism raises questions about motivations and political positioning of activist campaigns. This creates questions around grassroots activism and “top-down” powerful messages, the implications of which are still open for discussion.
